# Short-Term Effects of Dietary Selenomethionine Supplementation on Hepatic and Renal Transcriptomic Alterations Induced by Ochratoxin a in Broiler Chickens

**DOI:** 10.3390/toxins17090460

**Published:** 2025-09-12

**Authors:** Benjamin Kövesi, Szabina Kulcsár, Zsolt Ancsin, Márta Erdélyi, Erika Zándoki, Márk Tóth, Patrik Gömbös, Ágnes Freiler-Nagy, Krisztián Balogh, Miklós Mézes

**Affiliations:** 1 Department of Feed Safety, Institute of Physiology and Nutrition, Hungarian University of Agriculture and Life Sciences, Szent István Campus, H-2100 Gödöllő, Hungary; 2 HUN-REN-MATE Mycotoxins in the Food Chain Research Group, Hungarian University of Agriculture and Life Sciences, H-7400 Kaposvár, Hungary; 3 Agrobiotechnology and Precision Breeding for Food Security National Laboratory, Department of Physiology and Animal Health, Institute of Physiology and Nutrition, Hungarian University of Agriculture and Life Sciences, H-7400 Kaposvár, Hungary; 4 Department of Animal Hygiene, Herd Health and Mobile Clinic, University of Veterinary Medicine, H-1078 Budapest, Hungary

**Keywords:** ochratoxin A, selenomethionine, oxidative stress, glutathione redox system, aryl hydrocarbon receptor

## Abstract

Ochratoxin A (OTA), a mycotoxin commonly found in poultry feed, induces oxidative stress and disrupts redox homeostasis in vital organs such as the liver and kidneys. Selenium (Se), an essential trace element, may mitigate OTA-induced toxicity by supporting the antioxidant defense systems. This study investigated the short-term effects of dietary selenomethionine (SeMet) supplementation on OTA-induced oxidative and transcriptional responses in broiler chickens. Fifty-four 3-week-old birds were fed diets containing 2 mg/kg OTA, a target supplementation of 0.5 mg/kg Se (measured as 0.59 mg/kg as SeMet), or a combination of the two for five days. Liver and kidney samples were collected on Days 1 and 5 for biochemical and gene expression analyses. Exposure to OTA significantly modulated the expression of redox-sensitive transcription factors (*NRF2*, *KEAP1*), selenoproteins (*GPX3*, *GPX4*, *SELK*), and detoxification-related genes (*AHR*, *AHRR*, *CYP1A2*). SeMet alone enhanced selenoenzyme expression and antioxidant capacity, while co-exposure partially attenuated OTA-induced oxidative stress, resulting in more pronounced *NRF2* activation in the kidneys and *CYP1A2* induction in the liver. This is the first study to characterize the transcriptomic responses to OTA exposure in poultry within the first five days, providing novel insight into organ-specific mechanisms and emphasizing the epidemiological relevance of Se supplementation in mitigating the risk of feed contamination.

## 1. Introduction

Mycotoxins remain a significant challenge in poultry production, with ochratoxin A (OTA) being recognized as one of the most persistent and toxic contaminants. Produced by *Aspergillus* and *Penicillium* species, OTA is commonly detected in cereals, feed grains, and poultry products due to its chemical stability and resistance to conventional feed processing [1]. A central mechanism of OTA-induced toxicity is oxidative stress, which disrupts redox homeostasis through the overproduction of reactive oxygen and nitrogen species (ROS/RNS). This leads to cellular and nuclear damage, mitochondrial dysfunction, and ultimately impaired organ function in poultry [2].

OTA has been shown to alter the redox balance of the liver and kidneys by modulating key transcription factors and detoxification enzymes. In primary human hepatocytes, OTA was found to increase *AhR* mRNA levels and to induce several phase I enzymes, including CYP3A4, CYP2B6, CYP1A1 and CYP1A2. This suggests the activation of xenobiotic receptors such as AHR and PXR [3]. Shin et al. [4] further demonstrated that exposure to OTA (5–500 nM) triggers oxidative stress and apoptosis in HepG2 cells via the upregulation of phase I and phase II xenobiotic transformation enzymes. These responses were attenuated by siRNA-mediated knockdown of *AHR* and *NRF2*, confirming their central roles in OTA-induced hepatotoxicity.

In vivo findings support these results. Lee et al. [5] administered OTA (at doses ranging from 0.2 to 3 mg/kg body weight, five days/week for six weeks) to 7-week-old ICR mice. They observed oxidative kidney damage, elevated levels of ROS, malondialdehyde (MDA), and the activation of AHR, PXR, and their downstream detoxification targets. Similar effects were reported in OTA-treated HK-2 human kidney cells, including depletion of intracellular glutathione and enhanced transcription of phase I and II enzymes. Consistently, our previous work demonstrated that exposure to OTA (1 mg/kg feed for 21 days) significantly increased *NRF2* and *AHR* expression in the liver and kidneys of broiler chickens [6].

Despite regulatory limits in the EU (0.1 mg/kg feed for poultry; Commission Recommendation 2006/576/EC, amended 2016/1319/EC), OTA contamination remains a recurring problem. The most recent global survey by DSM-Firmenich (Satigny, Switzerland) [7] detected OTA in 8% of wheat grain samples (max. 928 ppb), 9% of corn kernels (max. 331 ppb) and 23% of finished feed samples (max. 173 ppb) in Europe. Similarly, Bonerba et al. [8] reviewed multiple studies showing that OTA levels in complete poultry feed often exceed the EU guidance value of 0.1 mg/kg, with maize being the ingredient most frequently affected. These findings highlight the continuing epidemiological relevance of OTA contamination and the need for effective mitigation strategies.

Selenium (Se) is an essential trace element that becomes biologically active when incorporated into selenoproteins, which are particularly involved in redox regulation and antioxidant defense. Among them, selenium-dependent glutathione peroxidases (GPxs), catalyze the reduction of hydrogen and lipid peroxides using reduced glutathione (GSH). supported by glutathione reductase (GSR), which maintains intracellular redox balance [9]. Selenomethionine has been widely reported to mitigate the toxic effects of several mycotoxins, including OTA, aflatoxin B1 (AFB1), T-2 toxin, and moniliformin [10]. In a poultry model, Li et al. [11,12] showed that 21 days of dietary exposure to OTA (50 µg/kg) resulted in oxidative stress and apoptosis in the liver and kidneys of 1-day-old broilers. Exposure to OTA suppressed the genes encoding antioxidant enzymes (*HO-1*, *GPx*, and *MnSOD*), downregulated *NRF2*, and upregulated pro-apoptotic markers (*CASPASE-3* and *BAX*). Co-administration of selenomethionine (0.4 mg/kg) restored redox balance, upregulated NRF2/KEAP1-related genes, and significantly alleviated OTA-induced organ damage.

Despite the growing body of evidence regarding the redox- and xenobiotic-disrupting effects of OTA, the early transcriptional responses in poultry following short-term exposure remain poorly characterized. Furthermore, while the protective role of selenium has been investigated in chronic settings, its influence on the initial activation of redox-sensitive transcription factors, particularly the NRF2/KEAP1 and AHR/AHRR pathways, has not been comprehensively studied in poultry.

Therefore, the aim of the study was to assess whether dietary supplementation with selenomethionine could mitigate the early oxidative and inflammatory effects of OTA in the liver and kidneys of broiler chickens after 5 days of exposure. Our focus was on the expression of glutathione-related antioxidant enzymes and transcription factors involved in redox and xenobiotic signaling in order to gain better understanding of the potential protective properties of selenium against OTA-induced toxicity.

## 2. Results

### 2.1. Clinical Findings

Throughout the study, no clinical signs of toxicity or mortality were observed in any of the experimental groups. Furthermore, body weight and the relative weights of the liver and kidneys remained unaffected, with no statistically significant differences detected between groups.

### 2.2. Effects of Se and OTA on Hepatic and Renal GSH Content and GPx Activity

**Figure 1:**
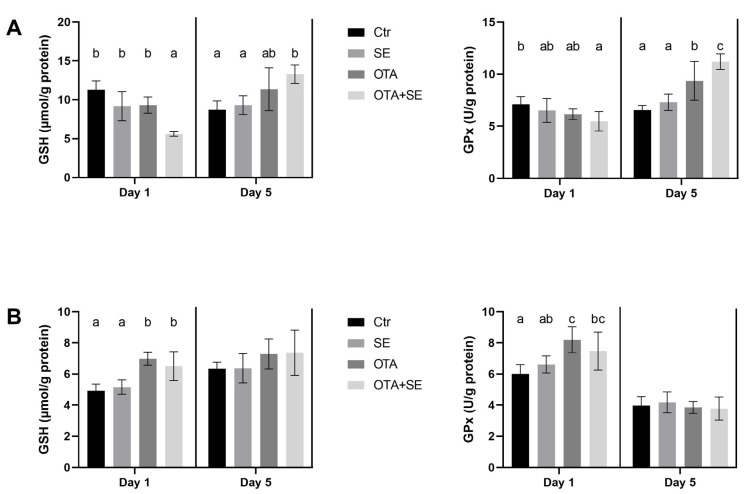
Effect of 5 days of OTA and SeMet on GSH content and GPx activity in the 10,000 g supernatant fraction of liver (**A**) and kidney (**B**) homogenates. Data are expressed as mean ± standard deviation (SD), *n* = 6; Ctr, control group; SE, Selenomethionine group (0.5 mg kg^−1^ feed); OTA, Ochratoxin A group (2 mg kg^−1^ feed); OTA+SE, Ochratoxin A (2 mg kg^−1^ feed) and Selenomethionine (0.5 mg kg^−1^ feed) group. Different superscripts in the same column mean a significant difference at *p* < 0.05, while the same letter indicates no significant difference between groups.

On Day 1, the glutathione (GSH) content of the 10,000 g supernatant fraction of liver homogenates was significantly lower in the OTA+SeMet group than in the SeMet and OTA groups (*p* < 0.05). On day 5 an opposite tendency was found. The highest GSH level was found in the OTA+SeMet group (Figure 1A). Conversely, GSH levels in kidney homogenates were significantly higher in the OTA and OTA+SeMet groups than in the control and SeMet groups on Day 1 (*p* < 0.05). However, there was no significant difference between the groups on day 5 (Figure 1B). Glutathione peroxidase (GPx) activity in the supernatant fraction of liver homogenate was significantly lower in the OTA+SeMet group than in the control on Day 1. However, by Day 5, GPx activity had increased significantly in both the OTA and OTA+SeMet groups relative to the control and SeMet groups (*p* < 0.05) (Figure 1A). In the supernatant fraction of kidney homogenates, GPx activity was significantly higher in the OTA and OTA+SeMet groups than in the control on Day 1. By Day 5, no significant differences in kidney GPx activity were observed among the exposure groups (Figure 1B).

### 2.3. Gene Expression Changes in Liver Tissue

Significant changes in were also observed in response to OTA, selenomethionine, and the two substances combined, as analyzed in liver tissue samples collected on Day 1 and 5.

#### 2.3.1. Oxidative Stress and Antioxidant Genes

**Figure 2:**
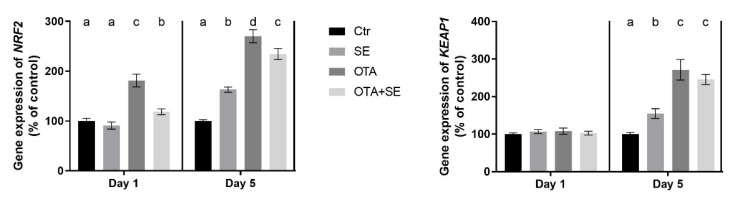
Effect of 5 days of OTA and SeMet on the relative expression of *NRF2* and *KEAP1* genes in the liver of broiler chickens; Data are presented as the mean percentage relative to control ± SD; *n* = 6; Ctr, control group; SE, Selenomethionine group (0.5 mg kg^−1^ feed); OTA, Ochratoxin A group (2 mg kg^−1^ feed); OTA+SE, Ochratoxin A (2 mg kg^−1^ feed) and Selenomethionine (0.5 mg kg^−1^ feed) group. Different superscripts in the same column mean a significant difference at *p* < 0.05, while the same letter indicates no significant difference between groups.

On Day 1, *NRF2* expression was significantly higher in the OTA and OTA+SeMet groups than in the control and SeMet groups (*p* < 0.05). On Day 5, *NRF2* expression was significantly higher in the SeMet, OTA and OTA+SeMet groups than in the control (*p* < 0.05), with the highest expression observed in the OTA group, followed by the OTA+SeMet and SeMet groups (Figure 2).

*KEAP1* expression showed no difference between the exposure groups on Day 1; however, on Day 5 the expression was significantly higher (*p* < 0.05) in all exposure groups compared to control (Figure 2).

**Figure 3:**
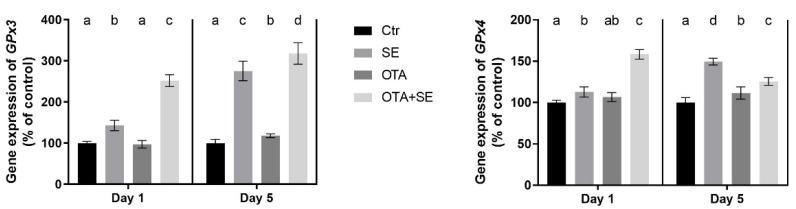
Effect of 5 days of OTA and SeMet on the relative expression of *GPX3* and *GPX4* genes in the liver of broiler chickens; Data are presented as the mean percentage relative to control ± SD; *n* = 6; Ctr, control group; SE, Selenomethionine group (0.5 mg kg^−1^ feed); OTA, Ochratoxin A group (2 mg kg^−1^ feed); OTA+SE, Ochratoxin A (2 mg kg^−1^ feed) and Selenomethionine (0.5 mg kg^−1^ feed) group. Different superscripts in the same column mean a significant difference at *p* < 0.05, while the same letter indicates no significant difference between groups.

*GPX3* expression was significantly higher in the Se and OTA+SeMet groups on Day 1 and 5 compared to the control and the OTA groups (*p* < 0.05). The OTA group also showed a moderate but statistically significant increase on Day 5 compared to the control group (Figure 3).

*GPX4* expression was significantly higher in the Se and OTA+SeMet groups than in the control on Day 1 (*p* < 0.05). By Day 5, expression was significantly higher in the SeMet, OTA and OTA+SeMet groups than in the control (*p* < 0.05), with the highest expression observed in the SeMet group, followed by the OTA+SeMet and OTA groups (Figure 3).

**Figure 4:**
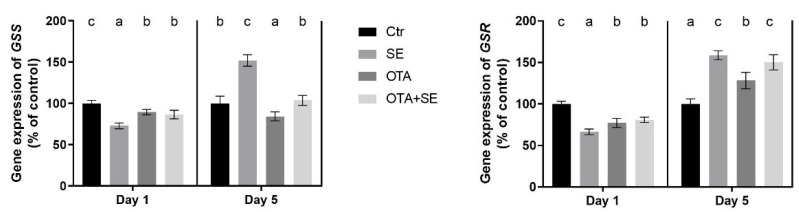
Effect of 5 days of OTA and SeMet on the relative expression of *GSS* and *GSR* genes in the liver of broiler chickens; Data are presented as the mean percentage relative to control ± SD; *n* = 6; Ctr, control group; SE, Selenomethionine group (0.5 mg kg^−1^ feed); OTA, Ochratoxin A group (2 mg kg^−1^ feed); OTA+SE, Ochratoxin A (2 mg kg^−1^ feed) and Selenomethionine (0.5 mg kg^−1^ feed) group. Different superscripts in the same column mean a significant difference at *p* < 0.05, while the same letter indicates no significant difference between groups.

On Day 1, *GSS* expression was significantly lower in all exposure groups (Se, OTA and OTA+SeMet) compared to the control (*p* < 0.05). By Day 5, *GSS* expression had increased significantly in the Se group, decreased significantly in the OTA group and was at the control levels in the OTA+SeMet group (Figure 4).

*GSR* expression followed a similar pattern to *GSS*. On Day 1, expression levels were significantly lower in all exposure groups than the control (*p* < 0.05). By Day 5, there was a significant increase in the *GSR* gene expression in the SeMet, OTA and OTA+SeMet groups compared to the control. However, there was a significant difference between the OTA group and the other two: OTA expression remained lower than that of both Se and OTA+SeMet, which did not differ significantly from each other (Figure 4).

**Figure 5:**
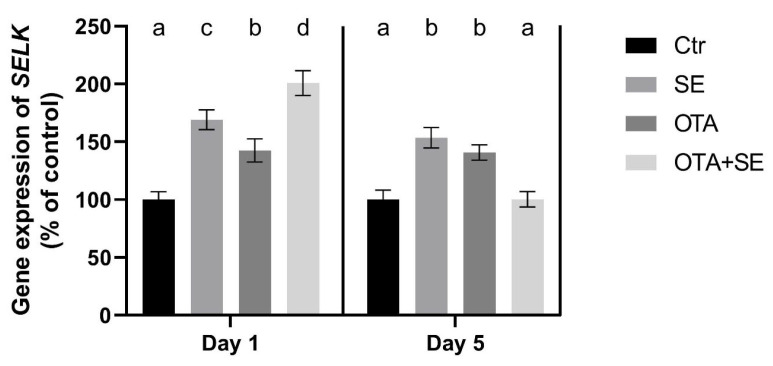
Effect of 5 days of OTA and SeMet on the relative expression of the *SELK* gene in the liver of broiler chickens; Data are presented as the mean percentage relative to control ± SD; *n* = 6; Ctr, control group; SE, Selenomethionine group (0.5 mg kg^−1^ feed); OTA, Ochratoxin A group (2 mg kg^−1^ feed); OTA+SE, Ochratoxin A (2 mg kg^−1^ feed) and Selenomethionine (0.5 mg kg^−1^ feed) group. Different superscripts in the same column mean a significant difference at *p* < 0.05, while the same letter indicates no significant difference between groups.

*SELK* expression was significantly higher in the SeMet and OTA+SeMet groups than in the control and OTA groups (*p* < 0.05) on Day 1, The OTA group also exhibited a moderate yet statistically significant increase relative to the control group. On Day 5, *SELK* expression remained significantly higher in the SeMet and OTA groups than in the control and OTA+SeMet groups (Figure 5).

#### 2.3.2. Detoxification Markers

**Figure 6:**
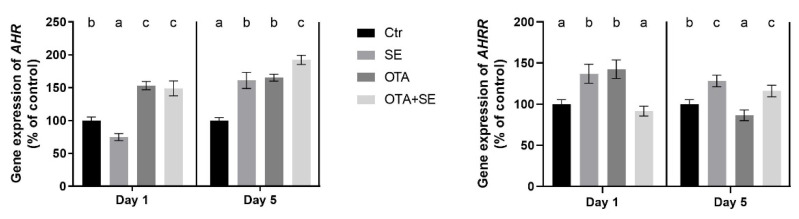
Effect of 5 days of OTA and SeMet on the relative expression of *AHR* and *AHRR* genes in the liver of broiler chickens; Data are presented as the mean percentage relative to control ± SD; *n* = 6; Ctr, control group; SE, Selenomethionine group (0.5 mg kg^−1^ feed); OTA, Ochratoxin A group (2 mg kg^−1^ feed); OTA+SE, Ochratoxin A (2 mg kg^−1^ feed) and Selenomethionine (0.5 mg kg^−1^ feed) group. Different superscripts in the same column mean a significant difference at *p* < 0.05, while the same letter indicates no significant difference between groups.

*AHR* expression was significantly higher in both the OTA and OTA+SeMet groups on Day 1 compared to the control (*p* < 0.05). This elevated expression persisted on Day 5, with all exposure groups showing significantly higher *AHR* expression than the control (Figure 6).

*AHRR* expression was significantly higher in both the SeMet and OTA groups on Day 1 compared to the control and OTA+SeMet (*p* < 0.05). On Day 5, the expression of *AHRR* was significantly lower in OTA but higher in SeMet and OTA+SeMet compared to control group (Figure 6).

**Figure 7:**
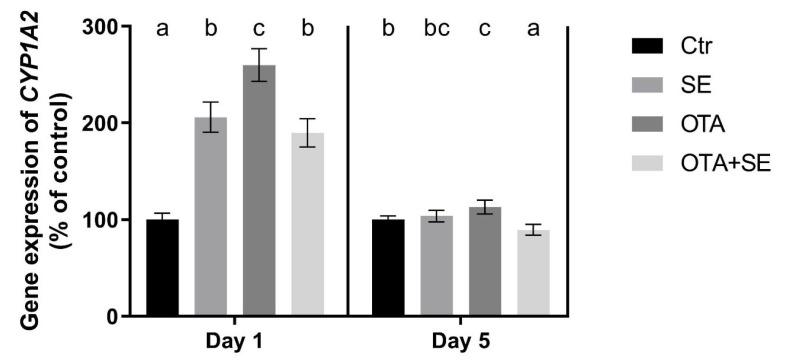
Effect of 5 days of OTA and SeMet on the relative expression of the *CYP1A2* gene in the liver of broiler chickens; Data are presented as the mean percentage relative to control ± SD; *n* = 6; Ctr, control group; SE, Selenomethionine group (0.5 mg kg^−1^ feed); OTA, Ochratoxin A group (2 mg kg^−1^ feed); OTA+SE, Ochratoxin A (2 mg kg^−1^ feed) and Selenomethionine (0.5 mg kg^−1^ feed) group. Different superscripts in the same column mean a significant difference at *p* < 0.05, while the same letter indicates no significant difference between groups.

*CYP1A2* expression was significantly higher in all exposure groups on Day 1 than in the control (*p* < 0.05). However, the SeMet and OTA+SeMet groups exhibited significantly lower expression than the OTA group. On Day 5, *CYP1A2* expression remained significantly higher in the OTA group than in the control, whereas the OTA+SeMet group showed significantly lower expression than both the OTA and control groups (Figure 7).

A summary of the gene expression changes observed in liver tissue is provided in Table 1.

### 2.4. Gene Expression Changes in Kidney Tissue

The expression of genes involved in oxidative stress and detoxification was analyzed in kidney tissue samples taken on Day 1 and Day 5 following dietary exposure to ochratoxin A (OTA; 2 mg/kg feed), selenomethionine (SeMet; 0.5 mg/kg feed), or a combination of the two.

#### 2.4.1. Oxidative Stress and Antioxidant Genes

**Figure 8:**
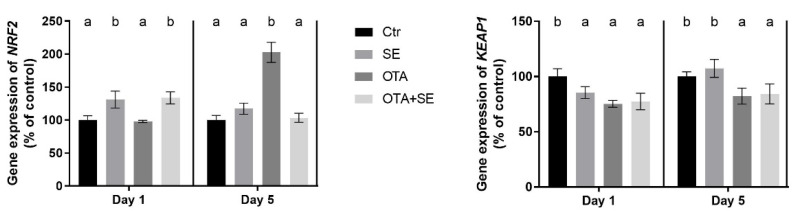
Effect of 5 days of OTA and SeMet on the relative expression of *NRF2* and *KEAP1* genes in the kidney of broiler chickens; Data are presented as the mean percentage relative to control ± SD; *n* = 6; Ctr, control group; SE, Selenomethionine group (0.5 mg kg^−1^ feed); OTA, Ochratoxin A group (2 mg kg^−1^ feed); OTA+SE, Ochratoxin A (2 mg kg^−1^ feed) and Selenomethionine (0.5 mg kg^−1^ feed) group. Different superscripts in the same column mean a significant difference at *p* < 0.05, while the same letter indicates no significant difference between groups.

*NRF2* expression was significantly higher in the Se and OTA+SeMet groups on Day 1 than in the control and OTA groups (*p* < 0.05). On Day 5, OTA significantly increased *NRF2* expression, whereas selenomethionine co-exposure restored it to control levels (Figure 8). *KEAP1* expression was significantly lower in all exposure groups compared to the control on Day 1 (*p* < 0.05) and in the OTA and OTA+SeMet groups on Day 5 as well (Figure 8).

**Figure 9:**
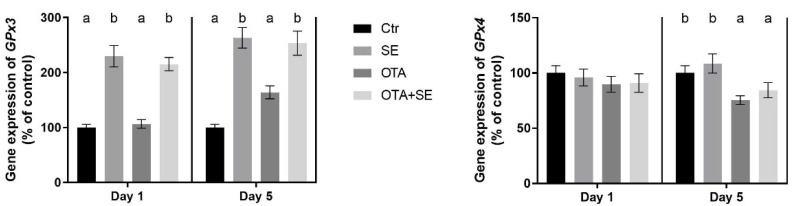
Effect of 5 days of OTA and SeMet on the relative expression of *GPX3* and *GPX4* genes in the kidney of broiler chickens; Data are presented as the mean percentage relative to control ± SD; *n* = 6; Ctr, control group; SE, Selenomethionine group (0.5 mg kg^−1^ feed); OTA, Ochratoxin A group (2 mg kg^−1^ feed); OTA+SE, Ochratoxin A (2 mg kg^−1^ feed) and Selenomethionine (0.5 mg kg^−1^ feed) group. Different superscripts in the same column mean a significant difference at *p* < 0.05, while the same letter indicates no significant difference between groups.

*GPX3* expression was significantly higher in the Se and OTA+SeMet groups on Day 1 and Day 5 compared to control and OTA groups (*p* < 0.05). Additionally, the OTA group showed a moderate but significant increase relative to control on Day 5 (Figure 9). *GPX4* expression was unaffected on Day 1. However, on Day 5, significant downregulation was observed in both the OTA and OTA+SeMet groups compared to the control (*p* < 0.05). This indicates that OTA suppresses *GPX4* expression, and that this suppression is not fully restored by selenomethionine (Figure 9).

**Figure 10:**
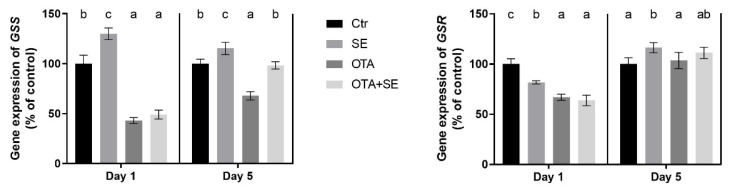
Effect of 5 days of OTA and SeMet on the relative expression of *GSS* and *GSR* genes in the kidney of broiler chickens; Data are presented as the mean percentage relative to control ± SD; *n* = 6; Ctr, control group; SE, Selenomethionine group (0.5 mg kg^−1^ feed); OTA, Ochratoxin A group (2 mg kg^−1^ feed); OTA+SE, Ochratoxin A (2 mg kg^−1^ feed) and Selenomethionine (0.5 mg kg^−1^ feed) group. Different superscripts in the same column mean a significant difference at *p* < 0.05, while the same letter indicates no significant difference between groups.

*GSS* expression was significantly higher in the SeMet group than in the control on both days (*p* < 0.05). By contrast, the OTA group exhibited significant suppression of *GSS* expression on Day 1 and 5. Interestingly, selenomethionine co-exposure (OTA+SeMet) restored *GSS* expression to control levels by Day 5 (Figure 10). *GSR* expression was significantly downregulated in all exposure groups compared to the control on Day 1 (*p* < 0.05). By Day 5, gene expression had increased significantly in the SeMet group, whereas the OTA and OTA+SeMet groups remained at levels comparable to the control (Figure 10).

**Figure 11:**
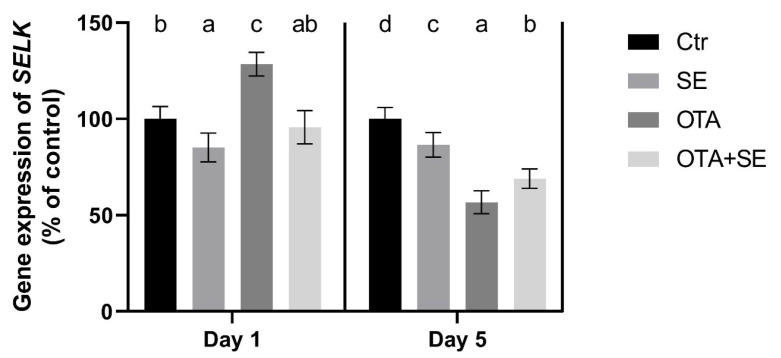
Effect of 5 days of OTA and SeMet on the relative expression of the *SELK* gene in the kidney of broiler chickens; Data are presented as the mean percentage relative to control ± SD; *n* = 6; Ctr, control group; Selenomethionine group (0.5 mg kg^−1^ feed); OTA, Ochratoxin A group (2 mg kg^−1^ feed); OTA+SeMet, Ochratoxin A (2 mg kg^−1^ feed) and Selenomethionine (0.5 mg kg^−1^ feed) group. Different superscripts in the same column mean a significant difference at *p* < 0.05, while the same letter indicates no significant difference between groups.

*SELK* expression was significantly upregulated in the OTA group on Day 1 compared to the control (*p* < 0.05). Selenomethionine co-exposure (OTA+SeMet) restored *SELK* expression to control levels. On Day 5, all exposure groups, including SeMet, OTA, and OTA+SeMet, showed significantly reduced *SELK* expression relative to the control group (*p* < 0.05) (Figure 11).

#### 2.4.2. Detoxification Markers

**Figure 12:**
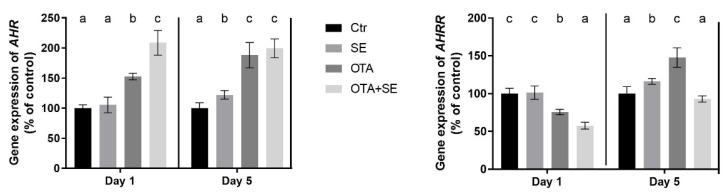
Effect of 5 days of OTA and SeMet on the relative expression of *AHR* and *AHRR* genes in the kidney of broiler chickens; Data are presented as the mean percentage relative to control ± SD; *n* = 6; Ctr, control group; SE, Selenomethionine group (0.5 mg kg^−1^ feed); OTA, Ochratoxin A group (2 mg kg^−1^ feed); OTA+SE Ochratoxin A (2 mg kg^−1^ feed) and Selenomethionine (0.5 mg kg^−1^ feed) group. Different superscripts in the same column mean a significant difference at *p* < 0.05, while the same letter indicates no significant difference between groups.

*AHR* expression was strongly induced by OTA on Day 1 (*p* < 0.05) and was further elevated in the OTA+SeMet group compared to all the other groups. This increased expression persisted on Day 5, with significantly higher levels observed in both the OTA and OTA+SeMet groups than in the control and SeMet groups (Figure 12). *AHRR* expression was significantly suppressed in the OTA and OTA+Se groups on Day 1 (*p* < 0.05), compared to the control and Se groups. However, a significant increase was observed on Day 5 in the Se and OTA groups compared to the control and OTA+Se groups (Figure 12).

**Figure 13:**
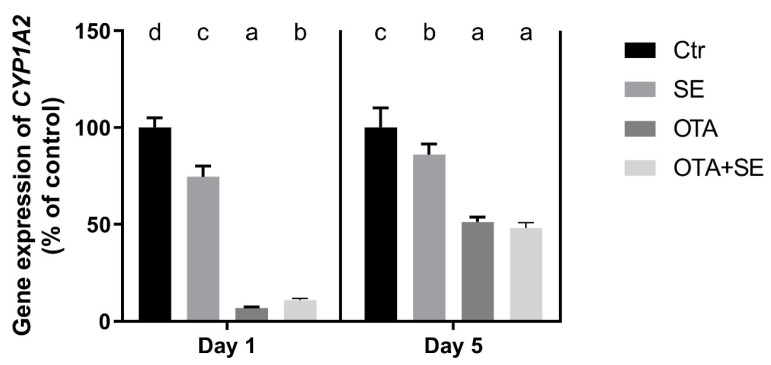
Effect of 5 days of OTA and SeMet on the relative expression of the *CYP1A2* gene in the kidney of broiler chickens; Data are presented as the mean percentage relative to control ± SD; *n* = 6; Ctr, control group; SE, Selenomethionine group (0.5 mg kg^−1^ feed); OTA, Ochratoxin A group (2 mg kg^−1^ feed); OTA+SE, Ochratoxin A (2 mg kg^−1^ feed) and Selenomethionine (0.5 mg kg^−1^ feed) group. Different superscripts in the same column mean a significant difference at *p* < 0.05, while the same letter indicates no significant difference between groups.

*CYP1A2* expression was significantly suppressed in the OTA group on both Day 1 and Day 5 compared to the control group (*p* < 0.05). A similar downregulation was observed in the OTA+SeMet group, suggesting that selenium co-exposure did not reverse the OTA-induced suppression (Figure 13).

A summary of the gene expression changes observed in kidney tissue is provided in Table 2.

## 3. Discussion

Contamination of poultry feed with mycotoxins, particularly ochratoxin A (OTA), remains a significant challenge in both intensive and small-scale poultry production. This is due to the widespread presence of OTA in cereal-based feeds and its remarkable stability during processing and storage [8]. Consequently, enhancing the endogenous defense mechanisms of poultry has emerged as a promising strategy for mitigating OTA-induced cellular damage [13]. Nutritional interventions, particularly the dietary supplementation of antioxidants such as selenium (Se), have demonstrated the ability to strengthen the redox balance and support detoxification pathways. By being incorporated into selenoproteins, selenium plays a pivotal role in regulating oxidative stress and maintaining cellular homeostasis [14]. Consequently, selenium and its organic form, selenomethionine [15], have the potential to mitigate the adverse effects of OTA-induced oxidative and inflammatory damage to vital organs such as the liver and kidneys when incorporated into the diet [16]. Several recent studies have demonstrated the protective effects of selenium against ochratoxin A (OTA)-induced toxicity in poultry. For example, Li et al. [11,12] showed that selenium yeast can alleviate OTA-induced nephrotoxicity and hepatotoxicity in chickens by activating the NRF2/KEAP1 and PI3K/AKT pathways. More recently, Tian et al. [17] showed that SeMet supplementation prevented OTA-induced renal injury in broilers by inhibiting ferroptosis. Meanwhile, Ren et al. [10] emphasized the importance of SeMet and sodium selenite in reducing OTA-induced apoptosis in mammalian kidney cells. While these studies consistently demonstrate that selenium supplementation counteracts OTA-induced oxidative stress and apoptosis, they have largely focused on exposure durations of 2–3 weeks. In contrast, our study uniquely demonstrates that transcriptional changes in NRF2/KEAP1 and AHR/AHRR signaling are evident within the first five days of OTA exposure. This highlights the temporal dynamics of OTA toxicity and the rapid onset of selenium-mediated modulation of redox and detoxification pathways.

### 3.1. Oxidative Stress Response Pathway

The results showed that exposure to OTA and SeMet supplementation activated the NRF2/KEAP1 redox sensitive pathway [18]. The expression of the *NRF2* gene suggested that SeMet had an inhibitory effect on the OTA-induced upregulation of *NRF2* gene expression. This indicates that SeMet can decrease the well-known OTA-induced oxidative stress as an antioxidant. However, this effect was organ-specific, being more pronounced in the kidney than in the liver. The effect of OTA in chickens was mainly observed in the kidneys, which could explain this difference [16]. On the other hand, *KEAP1* gene expression was lower in the kidneys of the OTA+SeMet group than in the control, meaning that the level of unbound NRF2 in the cells could increase, thereby activating the transcription of the genes encoding enzymes related to the glutathione redox system more effectively. In our experiment, the expression of both the *NRF2* and *KEAP1* genes was increased in the liver, particularly on Day 5 in all exposure groups. Under basal conditions, KEAP1 acts as an adaptor for CUL3-based E3 ubiquitin ligase activity. This promotes the constant ubiquitination and degradation of NRF2, thereby ensuring that NRF2 levels remain low. This system has been described as a ‘floodgate’ mechanism that prevents excessive NRF2 accumulation [19]. However, when the cell is exposed to oxidative stressors such as OTA, the ubiquitin ligase activity of the KEAP1-CUL3 complex is repressed without dissociation of the NRF2/KEAP1 complex [20]. Consequently, newly synthesized NRF2 avoids degradation, accumulates in the nucleus, and initiates the transcription of antioxidant response genes.

The expression of the *GPX3* and *GPX4* genes was upregulated by SeMet supplementation, but this effect was not related to the oxidative stress. As glutathione peroxidases (such as GPx3 and GPx4) are selenoenzymes, their activity and the initial upregulation of their gene expression increased. The differences in the gene expression and enzyme activity between the two glutathione peroxidases are possibly due to their different substrate specificity [21] and localization [22]. GPx3 is an extracellular enzyme that catalyzes the decomposition of hydrogen peroxide. In contrast GPx4 is a membrane-bound enzyme, whose substrates are lipid hydroperoxides. The results revealed that SeMet supplementation increased the GPx activity in both organs. However, this effect was observed earlier in the kidney (on Day 1) than in the liver (on Day 5). This difference can be explained by the different levels of GPx protein expression in the two organs. SeMet supplementation together with OTA exposure revealed that the *GPX3* gene responded more markedly to the OTA-induced redox changes than the *GPX4* gene did. This means that *GPX3* gene expression was more markedly upregulated by the combined effects of SeMet supplementation, OTA-induced oxidative stress and activation of the NRF2/KEAP1 pathway. *GPX3* was more sensitive than *GPX4* to combined OTA and selenium exposure. This suggests that it may be a better indicator of acute redox disturbances under selenium-responsive conditions.

Reduced glutathione (GSH) is a cofactor of glutathione peroxidases [22]; therefore, its cellular level can modify the activity of the enzyme. The results showed that the expression of glutathione synthetase (*GSS*) which is required for the GSH synthesis [23], was upregulated by SeMet supplementation in both organs due to an increase in de novo GPx synthesis, and consequently a higher requirement for the co-substrate GSH. OTA-induced redox changes downregulated the *GSS* in the kidney, which was partially compensated by Se supplementation. This effect can be explained by the impact of OTA exposure on the expression of *GPX3* and *GPX4* which did not increase significantly. The GSH content did not directly reflect the changes in gene expression which can be explained by the time-shift between gene and protein expression. Another important factor in maintaining cellular GSH levels is glutathione reductase (GSR), which reduces glutathione disulphide to reduced glutathione [24]. The expression of the *GSR* gene was downregulated on Day 1, but upregulated on Day 5. This suggests that redox changes activate the recycling of GSH as a delayed response. However, SeMet supplementation did not modify the effects caused by OTA exposure. This suggests that the expression of selenoenzyme glutathione peroxidases does not affect GSH recycling.

Selenoprotein K is a membrane- bound, selenium-containing protein that plays an important role in protecting against endoplasmic reticulum stress induced by oxidative stress [25]. Expression of the *SELK* gene in the liver showed an upregulation on Day 1 in all groups, including those exposed to both factors. However, this upregulation was not evident on Day 5 in the group exposed to OTA and supplemented with SeMet. This suggests that SeMet supplementation increases the expression of the *SELK* gene. The same effect was caused by exposure to OTA, which activates the antioxidant response due to oxidative stress, including defense against endoplasmic reticulum stress. Conversely, exposure to OTA, but not SeMet supplementation, increased *SELK* expression on Day 1. Downregulation was observed in all exposure groups in the kidney on Day 5. These results suggest that there is an organ-specific response to OTA-induced oxidative stress, and that SeMet activates the expression of selenoproteins, such as *SELK*, differently.

### 3.2. Detoxification Pathway

The first step in the detoxification pathway is the binding of a xenobiotic, such as OTA, to the aryl hydrocarbon receptor (AHR). The activity and nuclear translocation of the AHR are tightly regulated by the aryl hydrocarbon receptor repressor (AHRR), which acts as a negative regulator of AHR signaling via a feedback mechanism [26]. The results showed that the effect of OTA is manifested through this pathway, and that SeMet modulates it. OTA caused the *AHR* gene to be upregulated, and SeMet supplementation caused it to be downregulated. The effect of OTA was not modified by SeMet supplementation on Day 1, but upregulation was found in all exposure groups in the liver. In the kidneys, the changes showed some differences. OTA exposure was found to upregulate the *AHR* gene, and this effect was greater in the presence of SeMet supplementation. This suggests that SeMet activates the AHR receptor in the presence of OTA. In addition to this upregulation, a significant decrease in *AHRR* expression was observed in the kidneys on Day 1. On Day 5, OTA exposure caused *AHRR* gene expression to increase, but SeMet supplementation reduced this effect. *AHRR* expression exhibited a more intricate pattern in the liver. On Day 1, *AHRR* expression was significantly higher in the SeMet and OTA+SeMet groups than in the OTA group. This suggests that selenium may facilitate the feedback inhibition of AHR by inducing *AHRR* expression. By Day 5, the highest level of *AHRR* expression was observed in the SeMet group, followed by the OTA group. In contrast, it declined in the OTA+SeMet group that prolonged co-exposure may reduce AHRR-mediated repression and allow sustained AHR activation. These results suggest that OTA activates the AHR/AHRR system as a xenobiotic. Conversely, SeMet was found to have an inhibitory effect on AHR in mice. Based on this finding, SeMet has been proposed as a preventive antioxidant against xenobiotic-mediated oxidative stress [27].

Cytochromes, such as CYP1A2 are required for xenobiotic transformation, and their gene expression and activity are activated by the AHR pathway [28]. The overexpression of *CYP1A2* in the liver on Day 1 which is an early response, can be explained by the simultaneous overexpression of *AHR* and downregulation of *AHRR.* This effect was not observed on Day 5, indicating that the later response to OTA exposure is mediated by a different pathway. In contrast, OTA exposure caused marked downregulation of *CYP1A2* in the kidney, despite *AHR* upregulation. This is probably due to the fact that the liver is the main organ responsible for the transformation of xenobiotics, meaning that this pathway is more active and sensitive to them.

## 4. Conclusions

In conclusion, OTA induces oxidative stress in both the liver and kidneys, but the signaling pathways differ. In the liver, both the NRF2/KEAP1 and AHR/AHRR/cytochrome pathways are activated. In contrast, the AHR/AHRR/cytochrome pathway showed only moderate activation in the kidneys. Although selenomethionine supplementation activates the glutathione redox and xenobiotic transformation systems, changes in gene expression show that it does not fully counteract the effects of OTA. Future studies should extend the exposure period and integrate histopathological assessments. Furthermore, they should also explore whether dietary selenium supplementation could be translated into practical feed strategies to reduce the epidemiological risk of ochratoxin A in poultry production.

## 5. Materials and Methods

### 5.1. Animals and Treatments

A total of 54 three-week-old Cobb cockerels were used in the experiment. Of these, 6 birds served as an absolute control for the gene expression study and did not receive any experimental diet. The remaining 48 birds were randomly allocated into four exposure groups (*n* = 12 per group). The birds were housed in pens bedded with pine wood shavings under standard husbandry conditions, with ad libitum access to feed and water and a natural light regimen (15 h light: 9 h dark).

The short-term OTA exposure trial lasted for 5 days. This period was chosen based on our previous protocol in order to investigate the early molecular responses to OTA and selenium supplementation, before any aging-related physiological changes could interfere with the results. At 20 days of age, prior to allocation, all birds were weighed individually to ensure uniformity. The average initial body weight was 783.31 ± 82.08 g, with no more than a 5% difference in mean body weight across groups.

The experimental groups were as follows: Control: basal diet with no OTA contamination or selenium supplementation; Selenomethionine (SeMet): basal diet supplemented with 0.5 mg/kg selenium as selenomethionine (Selisseo^®^, Adisseo, Antony, France; 100% hydroxy-selenomethionine). Feed analysis confirmed an actual selenium concentration of 0.59 mg/kg; Ochratoxin A (OTA): basal diet contaminated with 2 mg/kg OTA; OTA+SeMet: basal diet containing 2 mg/kg OTA and supplemented with 0.5 mg/kg selenium as selenomethionine (measured value 0.59 mg/kg). The applied OTA concentration (2 mg/kg feed) represents a level characteristic of highly contaminated feed batches that may occasionally occur in practice. This elevated dose was deliberately chosen to reliably provoke measurable biochemical and transcriptional changes within the short 5-day exposure period.

At each time point (Day 1 and Day 5), six animals per group were euthanized via cervical dislocation. Liver and kidney samples were immediately collected, frozen in liquid nitrogen, and stored at −80 °C until further analysis for biochemical parameters and gene expression.

The basal diet contained 88.76% dry matter, 21.25% crude protein, 4.25% crude fiber, 1.10% calcium, 1.12% lysine, 0.38% methionine, 0.89% methionine + cysteine, 0.48% available phosphorus, 0.25% sodium, and 12.66 MJ/kg metabolizable energy. The diet excluded mycotoxin binders and coccidiostats.

The guidelines set by the European Communities Council Directive (86/609 EEC) were followed during the experiment. The protocol was approved by the Animal Welfare Committee of the Hungarian University of Agriculture and Life Sciences (MKK-TAKT-003/2019, 27 February 2019).

### 5.2. Mycotoxin Production

Ochratoxin A was produced by artificial inoculation of sterile ground corn substrate with *Aspergillus albertensis* strain SZMC 22107, obtained from the Microbiological Collection of the University of Szeged (SZMC). Following a suitable incubation period, the OTA-contaminated substrate was dried, ground, and incorporated into the experimental diets to achieve the desired final concentration.

OTA levels in the feed were confirmed using QuEChERS extraction (performed in triplicate), followed by liquid chromatography–mass spectrometry (LC-MS) analysis using a Shimadzu LCMS-2020 system (Shimadzu, Kyoto, Japan). The predicted and analytically confirmed OTA concentrations in the feed are presented in Table 3.

### 5.3. Determination of Selenium Concentration in Feed

Reagents of trace analytical grade were employed throughout the sample preparation procedure. Hydrogen peroxide (30 m/m%, AnalaR NORMAPUR), nitric acid (69 m/m%, Aristar), hydrochloric acid (37 m/m%, Aristar), and ethanol (AnalaR NORMAPUR) were sourced from VWR International Ltd. (Lutterworth, Leicestershire, UK). Ultrapure water with a resistivity of 18.2 MΩ/cm was generated using a Purite Select Fusion 160 BP purification unit (SUEZ Water Technologies and Solutions, Trevose, PA, USA).

To calibrate the inductively coupled plasma mass spectrometer (ICP-MS) (Perkin Elmer, Waltham, MA, USA), a multi-element calibration standard (Instrument Calibration Standard 2, TruQms; PerkinElmer, Shelton, CT, USA) was utilized. Internal standards were introduced through the Internal Standard Mix (TruQms); both standards were supplied by Perkin Elmer Inc. (Waltham, MA, USA). A hay powder reference material (BCR-129) obtained from Merck KGaA (Darmstadt, Germany) was used to prepare the quality control samples. The argon gas employed had a purity grade of 4.8 and was provided by Messer Hungarogaz Ltd. (Budapest, Hungary). Each sample, weighing 0.5 g, was placed into a CEM MARS XPreSS Teflon digestion vessel (CEM Corporation, Matthews, NC, USA) and combined with 5 mL of hydrogen peroxide and 5 mL of nitric acid. The mixture was subjected to microwave-assisted digestion using a CEM MARS6 system (CEM Corporation, Matthews, NC, USA) with the following settings: ramp duration of 35 min, final temperature of 200 °C, hold time of 50 min, microwave power of 1700 W, across 40 vessels. Upon completion of the digestion process, the contents were transferred and rinsed into 50 mL polypropylene (PP) tubes (Deltalab, Rubí, Barcelona, Spain). The final volume was adjusted to 25 mL with deionized water, then diluted fivefold in 12 mL PP tubes (Deltalab, Rubí, Barcelona, Spain) using the same water quality. Prior to ICP-MS analysis, each sample was spiked with 0.4 mL of ethanol and 100 µL of an internal standard solution, containing bismuth (Bi), germanium (Ge), and indium (In) at 1 µg/mL concentration. Quality control and blank samples were prepared identically. Between digestion runs, the Teflon vessels were cleaned with 0.15 M hydrochloric acid solution.

Elemental quantification was performed using a Perkin Elmer NEXION 2000 ICP-MS instrument (Perkin Elmer, Waltham, MA, USA) operated in helium KED mode. The following instrumental settings were applied: solid-state RF generator at 34.5 MHz (LumiCoil, free running), RF power of 1600 W, Meinhard Plus quartz nebulizer (type C, low internal volume), plasma gas flow rate of 15 L/min, auxiliary gas flow rate of 1.2 L/min, and nebulizer gas flow rate of 1.06 L/min. The helium cell gas flow was set to 5.6 mL/min (low) and 6.7 mL/min (high).

Table 4 presents the analytical isotopes measured, along with the internal standards selected for each analyte and the corresponding limits of detection (LOD). The LOD values were calculated as three times the standard deviation of the concentrations obtained for blank samples. The measured selenium concentrations in experimental diets are presented in Table 3.

### 5.4. Measurement of Reduced Glutathione Content and Glutathione Peroxidase Activity

The liver and kidney samples (0.4 g) were homogenized in physiological saline (0.65% (*w*/*v*) NaCl) at a 1:9 ratio. Reduced glutathione (GSH) content and glutathione peroxidase (GPx) activity was measured in the 10,000 g supernatant fraction of the homogenate. GSH concentration was determined by the method of Sedlak and Lindsay [29]. GPx activity was determined according to Lawrence and Burk [30] GSH content and GPx activity were calculated relative to the protein content of the supernatant fraction which was measured by the Folin–Ciocalteu phenol reagent (Merck, Darmstadt, Germany) [31].

### 5.5. Total RNA Extraction and Reverse Transcription

Total RNA was extracted from liver and kidney tissues (10 mg) using NucleoZOL reagent (Macherey-Nagel, Düren, Germany), following the manufacturer’s instructions. Residual genomic DNA was removed by treatment with DNase I (Thermo Fisher Scientific, San Jose, CA, USA). RNA concentration and purity were assessed using a NanoPhotometer (Implen, Munich, Germany), and samples with A260/A280 ratios > 2.0 were considered acceptable for downstream analysis. RNA integrity was verified via electrophoresis on a 1.5% agarose gel. Subsequently, 1 µg of high-quality RNA was reverse transcribed into complementary DNA (cDNA) using the High-Capacity cDNA Reverse Transcription Kit (Thermo Fisher Scientific), according to the manufacturer’s protocol.

### 5.6. Quantitative Real-Time Polymerase Chain Reaction

Quantitative real-time PCR (qPCR) was performed on a StepOnePlus™ Real-Time PCR System (Applied Biosystems™, Foster City, CA, USA) using PowerUp™ SYBR™ Green Master Mix (Thermo Fisher Scientific). Each 10 µL reaction contained 5.0 µL SYBR Green Master Mix, 0.2 µL each of forward and reverse primers (10 µM), 1.6 µL cDNA template, and 3.0 µL nuclease-free water.

A touchdown qPCR (TqPCR) protocol, adapted from Zhang et al. [32], was employed with minor modifications:

- Initial activation: 50 °C for 2 min, 95 °C for 10 min.
- Touchdown cycling: 4 cycles of 95 °C for 15 s, 64 °C for 30 s (decreasing 2 °C per cycle), and 72 °C for 30 s.
- Amplification: 36 cycles of 95 °C for 15 s, 58 °C for 30 s, 72 °C for 30 s.
- Melt curve: 95 °C for 15 s, 60 °C for 1 min, 95 °C for 15 s.

Each reaction was performed in triplicate and no-template controls (NTC) as well as reverse transcription minus (−RT) controls were included in each run to exclude contamination and genomic DNA amplification. Specificity was confirmed via melt-curve analysis. Primer efficiency (E) was calculated from the slope of standard curves generated using fivefold serial dilutions of pooled cDNA, using the formula: E = [10^(−1/slope) − 1] × 100% [33]. For normalization of gene expression, three commonly used reference genes (*GAPDH*, *β-actin*, and *RPL13*) were evaluated. Their expression stability was confirmed across all exposure groups, and the geometric mean of their relative expression values was used as the normalization factor, in accordance with MIQE guidelines All primers exhibited amplification efficiencies between 90% and 110%, with correlation coefficients (R^2^) > 0.99. Relative gene expression was calculated using the Pfaffl method [34]. Primer sequences and efficiency data are presented in Table 5.

### 5.7. Statistical Analysis

All data are expressed as means ± standard deviation (SD). Normality was assessed using the Shapiro–Wilk test, while Bartlett’s and Brown–Forsythe tests were used to assess homogeneity of variance. One-way analysis of variance (ANOVA) was performed using GraphPad Prism v9.0 (GraphPad Software, San Diego, CA, USA), followed by Tukey’s post hoc test for multiple comparisons. Differences were considered statistically significant at *p* < 0.05.

## Figures and Tables

**Table 1:** Summary of gene expression changes in liver tissue of broiler chickens exposed to OTA and/or supplemented with SeMet.

	Day 1			Day 5		
Gene/Marker	OTA Effect	SeMet Effect	OTA+SeMet Effect	OTA Effect	SeMet Effect	OTA+SeMet Effect
*NRF2*	↑ vs. Ctrl	—	↑ vs. Ctrl	↑ (highest)	↑	↑ (lower than OTA, higher than Ctrl)
*KEAP1*	—	—	—	↑ vs. Ctrl	↑ vs. Ctrl	↑ vs. Ctrl
*GPX3*	—	↑	↑	↑ (moderate)	↑	↑ (similar to Se)
*GPX4*	—	↑	↑	↑	↑ (highest)	↑
*GSS*	↓	↓	↓	↓	↑	— (restored to Ctrl)
*GSR*	↓	↓	↓	↑ (but lower than Se and OTA+SeMet)	↑	↑ (similar to Se)
*SELK*	↑ (moderate)	↑	↑	↑	↑	— (no difference from Ctrl)
*AHR*	↑	—	↑	↑	↑	↑
*AHRR*	↑	↑	— (lower than both)	↓	↑	↑
*CYP1A2*	↑ (highest)	↑ (lower than OTA)	↑ (lower than OTA)	↑	↑	↓ (lower than OTA and Ctrl)

Symbols: ↑ upregulation, ↓ downregulation, — no significant change.

**Table 2:** Summary of gene expression changes in kidney tissue of broiler chickens exposed to OTA and/or supplemented with SeMet.

	Day 1			Day 5		
Gene/Marker	OTA Effect	SeMet Effect	OTA+SeMet Effect	OTA Effect	SeMet Effect	OTA+SeMet Effect
*NRF2*	—	↑	↑	↑	—	— (restored to Ctrl)
*KEAP1*	↓	↓	↓	↓	—	↓
*GPX3*	—	↑	↑	↑ (moderate)	↑	↑ (similar to Se)
*GPX4*	—	—	—	↓	—	↓
*GSS*	↓	↑	↓	↓	↑	— (restored to Ctrl)
*GSR*	↓	↓	↓	—	↑	—
*SELK*	↑	—	— (restored to Ctrl)	↓	↓	↓
*AHR*	↑ (strong)	—	↑↑ (stronger than OTA)	↑	—	↑
*AHRR*	↓	↓	—	↑	↑	— (no increase)
*CYP1A2*	↓	—	↓	↓	—	↓

Symbols: ↑ upregulation, ↓ downregulation, — no significant change, ↑↑ strong induction.

**Table 3:** Measured OTA and Se concentration in the complete feed.

Group	Measured Se (mg/kg)	Measured OTA (mg/kg)
Control	0.07	not detected
SeMet	0.59	not detected
OTA	0.07	2.04 ± 0.13
OTA+SeMet	0.59	2.09 ± 0.23

**Table 4:** Measured isotope, internal standard and limit of detection.

Element	Isotope (*m*/*z*)	Internal Standard Isotope	LOD (mg/kg)
Selenium (Se)	78	Ge74	0.010

**Table 5:** Primer sequences and parameters.

	Genes	GenBank Accession No.	Primer Sequences, 5′-3′	Length, bp.	Efficiency, %
Endogenous control genes	*GAPDH*	NM_204305.1	F-TGACCTGCCGTCTGGAGAAA R-TGTGTATCCTAGGATGCCCTTCAG	98	92.64
	*BAC*	NM_205518.2	F-GACGAGATTGGCATGGCTTTATTT R-TAAGACTGCTGCTGACACCTTC	92	96.29
	*RPL13*	NM_204999.2	F-GCTTAAACTGGCGGGCATTAAC R-GGCTTGCAGTGACTCTGTAGAT	97	94.97
Nrf2-antioxidant pathway genes	*KEAP1*	KU321503.1	F-CATCGGCATCGCCAACTT R-TGAAGAACTCCTCCTGCTTGGA	113	99.74
	*NRF2*	NM_205117.1	F-TTTTCGCAGAGCACAGATAC R-GGAGAAGCCTCATTGTCATC	110	91.74
	*GPX3*	NM_001163232.2	F-ATCCCCTTCCGAAAGTACGC R-GACGACAAGTCCATAGGGCC	129	102.51
	*GPX4*	NM_001346448.1	F-AGTGCCATCAAGTGGAACTTCAC R-TTCAAGGCAGGCCGTCAT	203	91.03
	*GSS*	XM_425692.6	F-GTACTCACTGGATGTGGGTGAAGA R-CGGCTCGATCTTGTCCATCAG	196	104.84
	*GSR*	XM_015276627.2	F-CCACCAGAAAGGGGATCTACG R-ACAGAGATGGCTTCATCTTCAGTG	208	91.76
	*SELK*	NM_001025441.2	F-GAAGAGGGCCTCCAGGAAAT R-GAGCCATTGGTGGTGGACTAG	84	103.89
AhR signaling pathway genes	*AHR*	NM_204118.3	F-GAAGACGGGTGAGAGTGGAA R-CGCTTCCGTAGATGTTCTGC	171	99.20
	*AHRR*	NM_001201387.2	F-AGAACGGCACCATGAGGAAG R-CAGAGGTCCGGTTCTGCTTT	73	99.86
	*CYP1A2*	NM_205146.3	F-CATTACGGATGGGCAGAGTT R-GAAGTTCTTCAGGGCGTTCT	94	93.76

## Data Availability

The data presented in this study are available on request from the corresponding author. The data are not publicly available due to ongoing related research and institutional data management policies.

## Abbreviations

The following abbreviations are used in this manuscript:

OTA	Ochratoxin A
Se	Selenium
SeMet	Selenomethionine
ROS	Reactive Oxygen Species
RNS	Reactive Nitrogen Species
GSH	Reduced Glutathione
GPx	Glutathione Peroxidase
GPX3/GPX4	Glutathione Peroxidase 3/4
GSS	Glutathione Synthetase
GSR	Glutathione Reductase
NRF2	Nuclear Factor Erythroid 2–Related Factor 2
KEAP1	Kelch-like ECH-Associated Protein 1
AHR	Aryl Hydrocarbon Receptor
AHRR	Aryl Hydrocarbon Receptor Repressor
CYP1A2	Cytochrome P450 1A2
qPCR	Quantitative Polymerase Chain Reaction
ER	Endoplasmic Reticulum
SELK	Selenoprotein K
MDA	Malondialdehyde
ICR	Institute of Cancer Research (refers to mouse strain)
